# Effect of RNF113A deficiency on oxidative stress-induced NRF2 pathway

**DOI:** 10.1080/19768354.2024.2349758

**Published:** 2024-05-11

**Authors:** Namjoon Cho, Yong-Eun Kim, Yunkyeong Lee, Dong Wook Choi, Chungoo Park, Jung-Hwan Kim, Keun Il Kim, Kee K. Kim

**Affiliations:** a Department of Biochemistry, Chungnam National University, Daejeon, Republic of Korea; b Department of Biological Sciences, Sookmyung Women’s University, Seoul, Republic of Korea; c Division of Biotechnology, Korea University, Seoul, Republic of Korea; d School of Biological Science and Technology, Chonnam National University, Gwangju, Republic of Korea; e Department of Pharmacology, School of Medicine, Institute of Medical Science, Gyeongsang National University, Jinju, Republic of Korea

**Keywords:** RNF113A, X-linked trichothiodystrophy, reactive oxygen species, NRF2, stress granule

## Abstract

The ring finger protein 113A (RNF113A) serves as an E3 ubiquitin ligase and a subunit of the spliceosome. Mutations in the *RNF113A* gene are associated with X-linked trichothiodystrophy (TTD). However, the cellular roles of RNF113A remain largely unknown. In this study, we performed transcriptome profiling of RNF113A knockout (KO) HeLa cells using RNA sequencing and revealed the upregulation of NRF2 pathway-associated genes. Further analysis confirmed that the KO of RNF113A promotes nuclear localization of the NRF2 protein and elevates the mRNA levels of NRF2 target genes. RNF113A KO cells showed high levels of intracellular reactive oxygen species (ROS) and decreased resistance to cell death following H_2_O_2_ treatment. Additionally, RNF113A KO cells more sensitively formed stress granules (SGs) under arsenite-induced oxidative stress. Moreover, RNF113A KO cells exhibited a decrease in glutathione levels, which could be attributed to a reduction in *GLUT1* expression levels, leading to decreased glucose uptake reactions and lower intracellular glucose levels. These alterations potentially caused a reduction in ROS scavenging activity. Taken together, our findings suggest that the loss of RNF113A promotes oxidative stress-mediated activation of the NRF2 pathway, providing novel insights into RNF113A-associated human diseases.

## Introduction

Oxidative stress is a significant hazard to human health that affects cells through DNA damage, mitochondrial dysfunction, and lipid oxidation (Panieri and Santoro 2016; Xie et al. 2016 Sies and Jones 2020;). Cells have developed several defense mechanisms to protect against oxidative stress caused reactive oxygen species (ROS). When ROS levels are high in cells, NRF2 dissociates from KEAP1, thereby stabilizing it from proteasomal degradation and facilitating its localization to the nucleus (Li et al. 2018; Zheng et al. 2021; Suzuki et al. 2023). NRF2 binds to antioxidant response elements (AREs) and regulates the transcription of numerous genes involved in antioxidant activity, iron metabolism, redox homeostasis, and cell survival (Hirotsu et al. 2012; Xi et al. 2023). Another ROS mechanism involves glutathione peroxidases, which convert ROS into water *via* oxidation of two reduced glutathione (GSH) molecules and the production of oxidized glutathione (GSSG) (Cheung and Vousden 2022).

High intracellular ROS levels cause DNA damage and contribute to several human diseases including trichothiodystrophy (TTD) (Vermeulen et al. 2000). TTD are characterized by intelligent defects and sulfur-deficient brittle hair (Brooks et al. 2011). Mutations in the nucleotide excision repair-associated genes have been identified in patients with TTD (Itin et al. 2001). In a recent study, a nonsense mutation was identified in the *Ring Finger Protein 113A* (*RNF113A*) gene in patients with X-linked TTD (Corbett et al. 2015). RNF113A, a member of the ring finger family, encodes C3H4-and C3H1 type zinc finger domains that act as E3 ubiquitin ligases (Lear et al. 2017). Additionally, protein structure analysis identified RNF113A as a spliceosome component (Haselbach et al. 2018; Fica et al. 2019). However, the cellular effects of RNF113A on the oxidative stress regulatory pathways have not been well elucidated.

In this study, we knocked out (KO) *RNF113A* in HeLa cells and performed RNA sequencing. Further analysis revealed the impact of RNF113A on the defense mechanisms against oxidative stress. Overall, these results provide new insights into the pathology of RNF113A-associated human diseases.

## Materials and methods

### Cell culture and transfection

HeLa, a human cervical cancer cell line, was purchased from the American Type Culture Collection and cultured in Dulbecco's modified Eagle’s medium (DMEM; WELGENE, LM 001-05) supplemented with 10% fetal bovine serum (Gibco, 12483-020) and 1% penicillin–streptomycin (WELGENE, LS202-02). Cells were maintained at 37°C CO_2_ incubator in a humidified atmosphere.

To perform the RNF113A knock-down experiment, cells were seeded in the 6-well plates (3 × 10^5^ cells/well; SPL, 30006) and, then, transfected with 1 μg of siRNF113A (5ʹ-GCG TCT TCA ATC CAG CGA AAG AAT T-3ʹ) or non-targeting control siRNA (Bioneer, SN-1012) for 48 h using PolyMag reagent (Chemicell, 9003), according to the manufacturer’s instructions.

### Establishment of RNF113A KO HeLa cells

The optimized sgRNA (5ʹ-AGC GCC CGA GCA TCT ACG TG-3ʹ) was cloned into a px330 vector and transfected with 1 μg of it into HeLa cells using jetPEI reagent (Polyplus, 101000053), according to the manufacturer’s instructions. Four days after the transfection, the cells were seeded at a concentration of 0.5 cells per well in 96-well plates (SPL, 30096) to obtain a single-cell clone. The clones were tested by immunoblot analysis, and the RNF113A KO HeLa cell line was selected for further analysis.

### RNA extraction and RNA sequencing analysis

Total RNA was extracted using a Hybrid-R^TM^ RNA extraction kit (GeneAll, 305-101), according to the manufacturer’s instructions, and RNA quality was confirmed using a Bioanalyzer 2100 system (Agilent Technologies). To prepare the RNA libraries, 1 μg of total RNA was processed using the TruSeq Stranded mRNA LT Sample Prep Kit (Illumina, RS-122-9005DOC). The libraries were sequenced using Illumina NovaSeq and low-quality bases and adapter sequences were removed from the raw data using Trimmomatic (v.0.36). The clean reads were aligned to the human genome (hg38) using HISAT2 (v.2.2.1) with default parameters, and gene expression levels were quantified using a unique alignment to Cufflinks (v.2.2.1).

### Immunofluorescence

Cells were seeded in the 4-well chamber slides (3 × 10^4^ cells/well; Nunc, 154526), fixed using 4% paraformaldehyde for 10 min at 25°C, and permeabilized using 0.5% Triton X-100 in phosphate-buffered saline (PBS) for 15 min at 25°C. Subsequently, the cells were blocked using blocking buffer (5% goat serum, 1% bovine serum albumin, and 0.05% Tween 20 in PBS) for 1 h at 25°C. Primary antibodies were diluted with blocking buffer and added to blocked samples overnight at 4°C. Next, the primary antibodies were washed with PBS supplemented with 0.05% Tween 20 (PBS-T), and secondary antibodies and 4ʹ,6-diamidino-2-phenylindole (DAPI; Invitrogen, D1306; 1:1000) were treated for 1 h at 25°C. After washing with PBST, the samples were mounted with mounting solution (Invitrogen, P36930) and images were captured using an LSM 880 microscope equipped with an Airyscan (ZEISS). The antibodies used in this study are as follows: anti-NRF2 (1:100; Santa Cruz Biotechnology, sc-365949), anti-RNF113A (1:250; Thermo Fisher Scientific, PA5-51355), anti-G3BP1 (1:500; Santa Cruz Biotechnology, sc-365338), Goat anti-mouse IgG antibody conjugated to Alexa Fluor 488 (Thermo Fisher Scientific, A11001; 1:500), Goat anti-rabbit IgG antibody conjugated to Alexa Fluor 488 (Thermo Fisher Scientific, A11008; 1:500), Goat anti-mouse IgG antibody conjugated to Alexa Fluor 594 (Thermo Fisher Scientific, A11005; 1:500), and Goat anti-rabbit IgG antibody conjugated to Alexa Fluor 594 (Thermo Fisher Scientific, A11012; 1:500).

### Immunoblot analysis

Cells were seeded in 6-well plates (3 × 10^5^ cells/well) and lysed with Tris-Triton lysis buffer [10 mM Tris-HCl (pH 7.4), 100 mM NaCl, 1 mM EDTA, 1 mM EGTA, 1% Triton X-100, 10% glycerol, and 0.1% sodium dodecyl sulfate] supplemented with a protease inhibitor cocktail (Roche, 50855100). Lysates were subjected to the BCA assay (iNtRON, 21071) for protein quantification and denatured with Laemmli sample buffer (BIO-RAD, 1610737), supplemented with 5% β-mercaptoethanol. Samples were separated by SDS-PAGE and transferred to a nitrocellulose membrane (Merck, HATF00010). To block the membrane, 5% skim milk (BIO-RAD, 1706404) in PBS-T was treated for 1 h at 25°C. Primary antibodies were diluted in skim milk-based blocking buffer and added to blocked membrane overnight at 4°C. Next, the membrane was washed using PBST and treated with secondary antibodies for 1 h at 25°C. Protein signals were detected using WSE-6200H LuminoGraph II (ATTO). The antibodies used in this study are as follows: anti-RNF113A (1:1000; Thermo Fisher Scientific, PA5-51355), anti-NRF2 (1:100; Santa Cruz Biotechnology, sc-365949), anti-GAPDH (1:2,000; Meridian Bioscience, H86045P).

### Quantitative real-time PCR

To synthesize the cDNA, 1 μg of total RNA was reverse transcribed with M-MLV reverse transcriptase (Promega, M170B), RNase inhibitor (Enzynomics, M007S), dNTPs, and random hexamer for 1 h at 50°C. To assess the mRNA expression levels, quantitative real-time PCR (qRT-PCR) was performed using SYBR Green PCR Master Mix (GENETBIO, Q-9200) on an AriaMx real-time system (Agilent Technologies). Primers used in this study are as follows: SCL7A11, 5ʹ-TGC TGG GCT GAT TTA TCT TCG-3ʹ and 5ʹ-GAA AGG GCA ACC ATG AAG AGG-3ʹ; HMOX1, 5ʹ-CAG GCA GAG AAT GCT GAG TTC-3ʹ and 5ʹ-GAC TGG GCT CTC CTT GTT GC-3ʹ; FTH1, 5ʹ-CGC CTC CTA CGT TTA CCT GTC-3ʹ and 5ʹ-AGC ATG TTC CCT CTC CTC ATG-3ʹ; FTL, 5ʹ-ATT TGT ACC TGC AGG CCT CC-3ʹ and 5ʹ-CCC ACT CAT CTT CAG CTG GC-3ʹ; GLUT1, 5ʹ-GGA CCT CAA ATT TCA TTG TGG GC-3ʹ and 5ʹ-GGT GAA GAT GAA GAA CAG AAC CAG-3ʹ; β-actin, 5ʹ-TCA CCC ACA CTG TGC CCA TCT ACG A-3ʹ and 5ʹ-CAG CGG AAC CGC TCA TTG CCA ATG G-3ʹ.

### Intracellular ROS analysis

Cells, seeded in 6-well plates (3 × 10^5^ cells/well), were trypsinized and incubated with 3 μM 2,7-dichlorofluorescin diacetate (DCF-DA; Sigma-Aldrich, D6883) in Hank's Balanced Salt Solution (HBSS; WELGENE, LB003-02) for 30 min at 37°C. DCF-DA fluorescence from 10,000 cells was measured using a BD FACSCanto II instrument (BD Biosciences). Data were processed using FlowJo software.

### Cell viability

Cells were seeded in 96-well plates (5000 cells/well) and treated with various concentration of H_2_O_2_ for 24 h. To analyze the cell viability, 20 μL of MTS solution (Promega, G3581) was added to wells and incubated for 30 min at 37°C. The absorbance was measured at 490 nm using a SpectraMax ABS Plus microplate reader (Molecular Devices). The absorbance of the MTS solution in cell-free medium was used as a baseline for absorbance correction.

### ^13^C-labeled Glucose tracing assay

HeLa cells were seeded in 6-well plates (3 × 10^5^ cells/well) for 24 h. Subsequently, the medium was washed twice with PBS and replaced with a glucose-free medium (WELGENE, LM 001-79) supplemented with 4500 mg/L [U^13^C]-D-glucose (Sigma-Aldrich, 660663). After incubation for 6 h, the radioactivity was measured using a Tri-Carb Liquid Scintillation Analyzer (Revvity).

### Intracellular glucose level measurement

HeLa cells were seeded in 90 mm culture plates (1.5 × 10^6^ cells/plate; SPL, 20100) for 24 h and were, then, trypsinized and centrifuged at 300 *g* for 5 min. Subsequently, the cells were washed twice with ice-cold isotonic saline solution (0.9% NaCl), and 350 μL of ice-cold 80% methanol were added. To obtain metabolites, the cells were sonicated with 10 s on/off cycle for 10 min at 4°C on BioRuptor (Diagenode) and then centrifuged for 10 min at 13,000 *g* at 4°C. The supernatants were transferred into a new tube and dried using vacuum centrifuge overnight at 4°C. The dried metabolites were suspended in pyridine (Sigma-Aldrich, 270970) supplemented with 10 mg/ml methoxylamine (Sigma-Aldrich, 225517), incubated for 30 min at 37°C, and derivatized with 70 μL of MTBSTFA (Sigma-Aldrich, 375934) for 1 h at 70°C. Intracellular glucose levels were analyzed by GC-MS with an Agilent 7890 B gas chromatograph coupled with a 5977 B mass-selective detector.

### GSH and GSSG measurement

HeLa cells were seeded in 96-well plates (5000 cells/well), and then treated with 500 μM H_2_O_2_ for 30 min. To quantify intracellular GSH and GSSG levels, a GSH/GSSG-Glo assay (Promega, V6611) was performed according to the manufacturer’s instructions.

## Results

### RNF113A deficiency enhances mRNA level of NRF2 target genes

**Figure 1.**
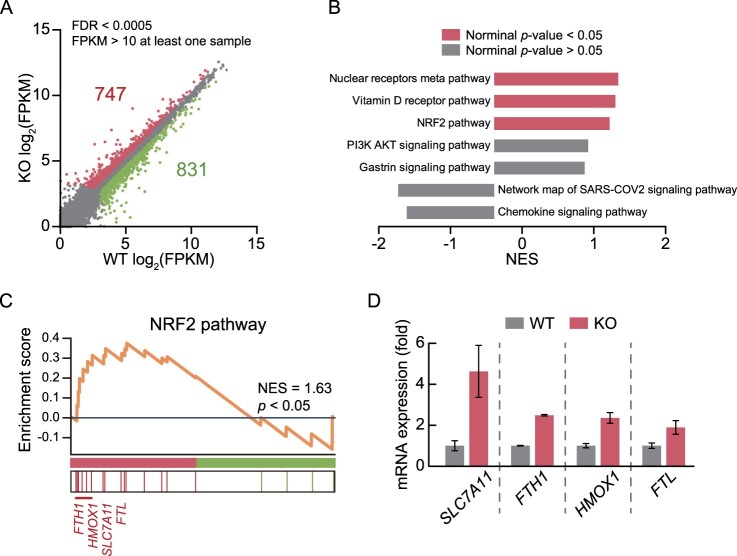
Transcriptome analysis reveals upregulation of NRF2 pathway-associated genes in RNF113A KO cells. (A) Scatter plot displaying gene expression of RNF113A KO and WT cells. Colored dots indicate DEGs (Upregulated genes = green; downregulated genes = red) in RNF113A KO cells compared to WT cells. (B) Bar graph representing significantly enriched terms (NES > 1 or < −1) from GSEA utilizing the WikiPathways gene set for DEGs. (C) GSEA enrichment plot of NRF2 pathway for DEGs. (D) mRNA expression levels of NRF2 pathway target genes. Columns represent the relative mRNA expression levels analyzed by FPKM values of RNA sequencing data from WT and RNF113A KO cells.

To evaluate the effects of RNF113A loss-of-function on gene expression, we deleted the *RNF113A* gene in HeLa cells using the CRISPR-Cas9 system. Subsequently, we performed RNA sequencing and found 1578 differentially expressed genes (DEGs) in *RNF113A* knockout (KO) cells compared to those in RNF113A wild-type (WT) cells (Figure 1A). Next, we clustered the DEGs using gene set enrichment analysis (GSEA) based on the WikiPathways gene set (Agrawal et al. 2024). The results showed that genes associated with the nuclear receptor meta pathway, Vitamin D receptor pathway, and NRF2 pathway were upregulated in RNF113A KO cells (Figure 1B and C). Among these pathways, current research has focused on the NRF2 pathway, which is a major pathway involved in the protective response against cellular stress, especially oxidative stress. We further confirmed that *FTH1*, *HMOX1*, *SLC7A11*, and *FTH*, the main NRF2 pathway target genes, were significantly upregulated in RNF113A KO HeLa cells (Figure 1D) (Kerins and Ooi 2018). We found that RNF113A KO affected the mRNA expression of NRF2 pathway-associated genes.

We also conducted a comprehensive analysis of alternative splicing in RNF113A KO cells compared to WT cells, found 1865 alternative splicing events in 1341 genes (Supplementary Figure 1A and 1B). Further gene ontology analysis of alternatively spliced genes in RNF113A KO cells revealed the top 6 significantly enriched terms (Supplementary Figure 1C). These results may be useful for understanding the role of RNF113A in cells.

### RNF113A deficiency promotes the NRF2 pathway

**Figure 2.**
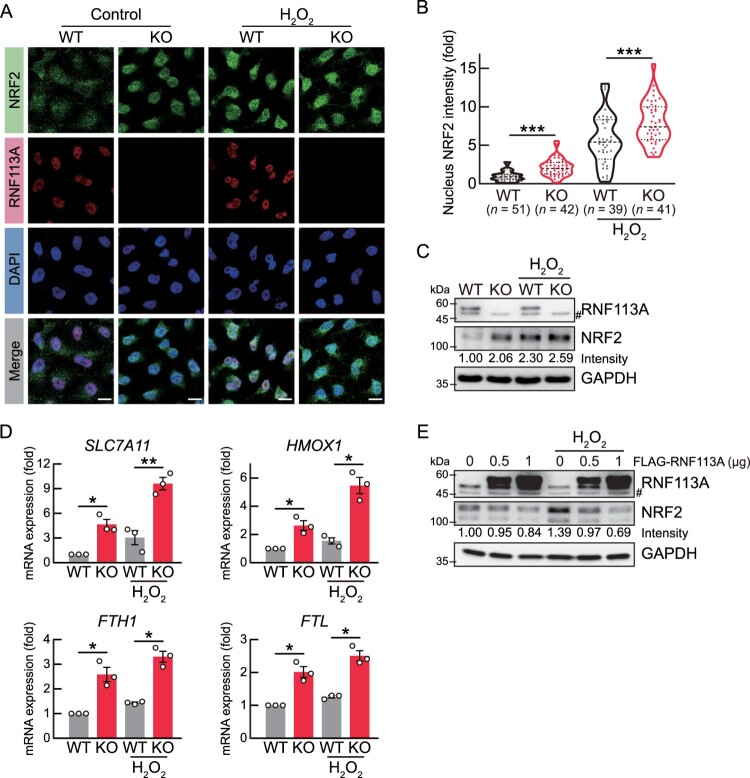
RNF113A deficiency increases NRF2 pathway. (A) Immunofluorescence analysis of NRF2 (green) and RNF113A (red) proteins with DAPI for nuclei (blue). Scale bars = 10 μm. (B) Violin plot representing the fluorescence intensities of NRF2 protein in the nucleus of each cell from immunofluorescence images. Nucleus NRF2 intensities were quantified from five independent immunofluorescence images. (C) Immunoblot analysis showing the NRF2 protein levels in RNF113A KO cells. WT or RNF113A KO cells were treated with 500 μM H_2_O_2_ for 30 min. #, non-specific protein band. (D) Bar graph showing the mRNA expression levels of NRF2 target genes. Cells were treated with 500 μM H_2_O_2_ for 6 h and then subjected to qRT-PCR analysis. (E) Immunoblot analysis of NRF2 protein levels. HeLa cells were transfected with mock or FLAG-RNF113A expression vectors for 24 h and then treated with 500 μM H_2_O_2_ for 30 min. *p*-values were calculated using two-tailed Student’s *t*-test. *p < 0.05, **p < 0.01, ***p < 0.001.

NRF2 translocates from the cytosol to the nucleus in response to oxidative stress and affects the expression of a wide range of genes involved in cellular protection. Therefore, the upregulation of NRF2 pathway-associated genes in RNF113A KO cells prompted us to analyze the NRF2 localization in these cells. We treated WT and RNF113A KO cells with 500 μM of H_2_O_2_ for 30 min and then performed immunofluorescence analysis using NRF2 and RNF113A antibodies (Figure 2A). This result revealed that RNF113A KO induced nuclear localization of NRF2 (Figure 2B). Moreover, we confirmed increased nuclear localization of the NRF2 protein in both WT and RNF113A KO cells after H_2_O_2_ treatment, and this nuclear NRF2 localization was significantly higher in RNF113A KO cells compared to WT cells. Under non-stress conditions, KEAP1 protein induces protein degradation of NRF2 *via* proteasomal degradation (Katoh et al. 2005; Cuadrado et al. 2019). Next, we analyzed NRF2 protein level in RNF113A KO cells using immunoblot analysis. The results showed that NRF2 protein levels were higher in RNF113A KO cells than in WT cells regardless of H_2_O_2_ treatment (Figure 2C). To assess the effects of nuclear NRF2 localization following exposure to H_2_O_2_ in RNF113A KO cells, we analyzed the mRNA levels of NRF2 target genes in H_2_O_2_-treated RNF113A KO cells and found significant increases in the mRNA expression levels of *SLC7A11*, *HMOX1*, *FTH1*, and *FTL* compared to those in H_2_O_2_-treated WT cells (Figure 2D). This result also showed that the mRNA expression levels of these NRF2 target genes were higher in RNF113A KO cells compared to H_2_O_2_-treated WT cells. Conversely, the intensity of nucleus NRF2 protein was higher in H_2_O_2_-treated WT cells than RNF113A KO cells. These differences suggest that RNF113A KO cells consistently activate the NRF2 pathway, leading to the accumulation of transcripts of NRF2 target genes. Finally, we confirmed that RNF113A overexpression in HeLa cells reduced NRF2 protein levels (Figure 2E). Collectively, these results suggested that RNF113A deficiency increases the transcriptional activities of NRF2 protein.

### RNF113A deficiency increases oxidative stress

**Figure 3.**
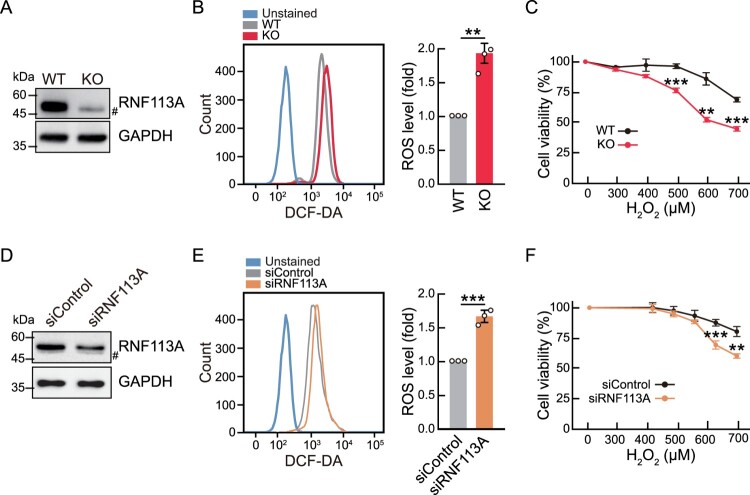
RNF113A deficiency increases intracellular ROS levels. (A) Immunoblot analysis confirming KO of RNF113A. (B) Intracellular ROS levels in WT and RNF113A KO cells. Cells were stained with DCF-CA and fluorescence was analyzed by flow cytometer (left). The bar graph (right) shows the mean values of ROS levels in WT or RNF113A KO cells. (C) Cell viability analysis of WT and RNF113A KO cells after H_2_O_2_ treatment. Cells were treated with H_2_O_2_ for 24 h, and cell viability was measured using the MTS assay. (D) Immunoblot analysis showing the knock down of RNF113A by siRNA transfection. (E) Intracellular ROS levels in siControl or siRNF113A transfected HeLa cells (left). The bar graph (right) shows mean values of ROS levels. (F) Cell viability analysis of siControl or siRNF113A transfected HeLa cells after H_2_O_2_ treatment. Cells were transfected with siRNAs for 48 h, followed by treatment with H_2_O_2_ for 24 h. Cell viability was measured using MTS assay. *p*-values were calculated using two-tailed Student’s *t*-test. **p < 0.01, ***p < 0.001.

Oxidative stress is the main activator of the NRF2 pathway. Given the increase in the activity of the NRF2 pathway in RNF113A KO cells, we analyzed oxidative stress in RNF113A KO cells. To assess intracellular ROS levels, we stained WT and RNF113A KO cells with DCF-DA dye and analyzed the fluorescence by flow cytometry (Figure 3A, B). These results revealed that cells with RNF113A KO exhibited high ROS levels than WT cells. Subsequently, we treated WT and RNF113A KO cells with various concentrations of H_2_O_2_ and measured cell viability using MTS assay (Figure 3C). RNF113A KO cells were significantly sensitized to H_2_O_2_ exposure compared to WT cells. We further investigated the oxidative stress response induced by the RNF113A knockdown (Figure 3D). DCF-DA analysis revealed that knockdown of RNF113A by siRNA transfection in HeLa cells increased intracellular ROS levels (Figure 3E). Moreover, the knockdown of RNF113A in HeLa cells sensitized them to H_2_O_2_-induced cytotoxicity (Figure 3F). These results revealed that RNF113A deficiency promotes oxidative stress, which may lead to the activation of the NRF2 pathway.

### RNF113A deficiency promotes stress granule (SG) formation

**Figure 4.**
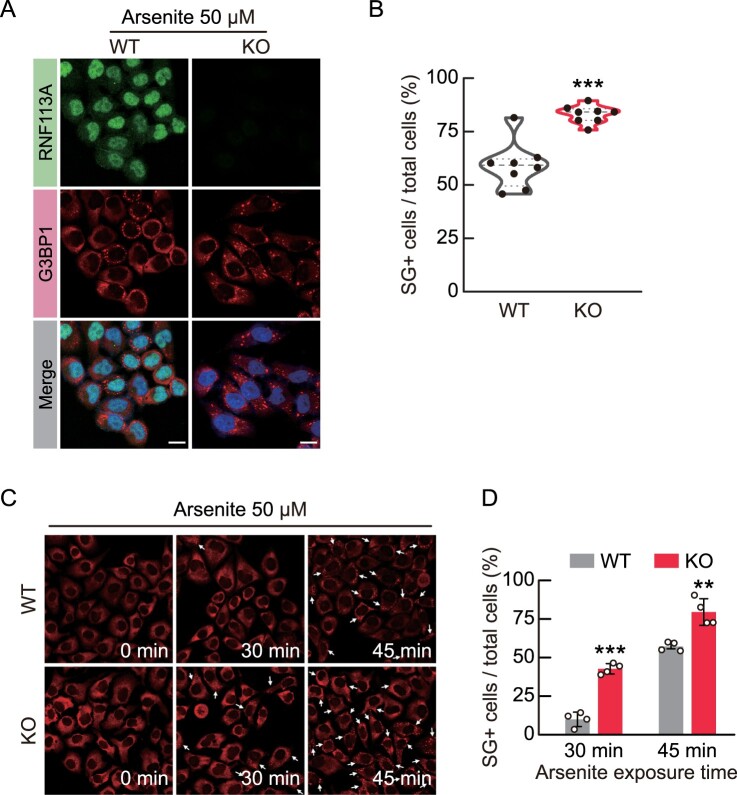
RNF113A KO increases sensitivity to arsenite-induced SG formation. (A) SG formation in WT and RNF113A KO cells upon arsenite exposure. Cells were treated with arsenite for 40 min and immunofluorescence analysis was performed using anti-RNF113A (green) and anti-G3BP1 (red) antibodies with DAPI for nuclei (blue). Scale bars = 10 μm. (B) Violin plot presenting proportion of SG-positive (SG+) cells from each immunofluorescence image. (C) Representative immunofluorescence images of G3BP1 (red) in cells at the indicated time point after 50 μM arsenite treatment. (D) Proportion of SG-positive cells from each immunofluorescence images. *p*-values were calculated using two-tailed Student’s *t*-test. **p < 0.01, ***p < 0.001.

To protect mRNA from various cellular stressors, including oxidative stress, G3BP1 acts as a scaffold and seed protein, aggregating RNA and other proteins to form SGs (Park et al. 2017). Therefore, we investigated whether RNF113A KO affected SG formation. Arsenite is a well-known SG inducer, which induces oxidative stress (Hu et al. 2020). We treated WT and RNF113A KO cells with arsenite and performed immunofluorescence using RNF113A and G3BP1 antibodies (Figure 4A). RNF113A KO cells exhibited a higher frequency of SG-positive cells following arsenite treatment compared to WT cells (Figure 4B). Moreover, we observed a high sensitivity of SG formation in RNF113A KO cells after 30 and 45 min of arsenite exposure (Figure 4C, D). Overall, these results suggest that RNF113A deficiency increases the sensitivity to arsenite-induced SG formation.

### RNF113A deficiency decreases glutathione level

**Figure 5.**
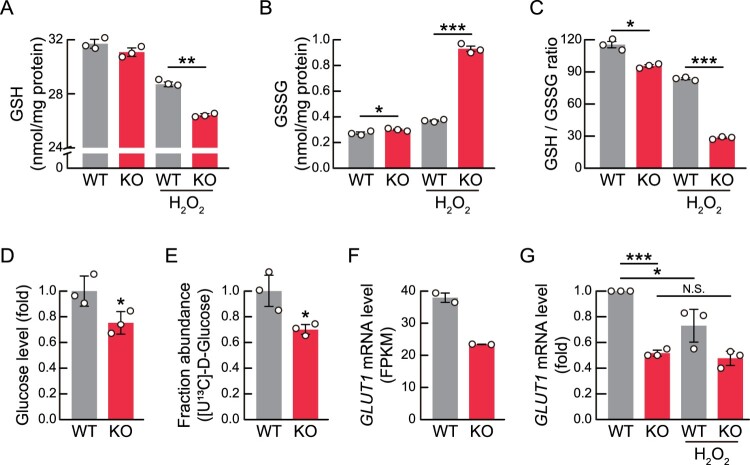
GSH levels are decreased in RNF113A KO cells. (A-C) Bar graphs representing intracellular GSH (A) and GSSG (B) levels, and ratios of GSH to GSSG (C) in WT or RNF113A KO cells, treated with H_2_O_2_ for 30 min. (D) Bar graph showing the intracellular glucose levels of WT and RNF113A KO cells. (E) Glucose uptake activities of WT and RNF113A KO cells. The cells were treated with ^13^C-labeled glucose for 6 h. (F) *GLUT1* mRNA expression levels of WT and RNF113A KO cells. The bar graph shows relative *GLUT1* FPKM values from RNA sequencing data. (G) *GLUT1* mRNA expression levels by H_2_O_2_ treatment in RNF113A KO cells. Cells were treated with 500 μM H_2_O_2_ for 6 h and then subjected to qRT-PCR analysis. *p*-values were calculated using two-tailed Student’s *t*-test. *p < 0.05, **p < 0.01, ***p < 0.001. N.S. = not significant.

To protect against ROS accumulation, cells possess antioxidant systems, including the glutathione antioxidant pathway. Glutathione peroxidases convert GSH into GSSG, and H_2_O_2_ into H_2_O (Cheung and Vousden 2022). Thus, the GSH level and the ratio of GSH to GSSG are crucial factors for protection against oxidative stress. Given that RNF113A KO increased intracellular ROS levels, we investigated GSH and GSSG levels in RNF113A KO and WT cells. We found that H_2_O_2_-treated RNF113A KO cells exhibited lower GSH levels than H_2_O_2_-treated WT cells (Figure 5A). Furthermore, GSSG levels were significantly higher in H_2_O_2_-treated RNF113A KO cells than in H_2_O_2_-treated WT cells (Figure 5B). The ratio of GSH to GSSG was lower in RNF113A KO cells compared than in WT cells regardless of H_2_O_2_ treatment (Figure 5C).

Cells recycle GSSG back into GSH, which is utilized for ROS scavenging, by oxidizing NADPH synthesized through the pentose phosphate pathway which utilizes glucose as the main source (Jeong et al. 2023). To reveal the reduction in GSH levels by RNF113A KO, we examined intracellular glucose levels by extracting metabolites from WT and KO cells and then performed LC-MS/MS analysis (Figure 5D). We found that the intracellular glucose levels were lower in RNF113A KO cells compared to WT cells. Isotope tracing revealed that the uptake of ^13^C-labeled glucose was reduced in RNF113A KO cells than in WT cells (Figure 5E). Furthermore, we confirmed from RNA sequencing that *GLUT1*, the glucose transporter gene, mRNA levels were significantly decreased in RNF113A KO cells compared to WT cells (Figure 5F). Given the increase in oxidative stress in RNF113A KO cells, we examined *GLUT1* mRNA expression levels in response to H_2_O_2_ treatment, it confirmed that treatment with 500 μM of H_2_O_2_ significantly reduced the *GLUT1* mRNA levels in WT cells, but RNF113A KO cells showed no significant difference in *GLUT1* mRNA levels with H_2_O_2_ treatment (Figure 5G). This result suggest that increased intracellular ROS levels in RNF113A KO cells could promotes the downregulation of GLUT1 expression, and it could further reduces ROS scavenging activities by reducing GSH levels. Overall, our results suggested that RNF113A deficiency decreased intracellular glucose levels by reducing glucose uptake, potentially leading to oxidative stress.

## Discussion

In this study, we analyzed global transcriptome alterations by KO of RNF113A and revealed the upregulation of NRF2 pathway target genes. H_2_O_2_ treatment significantly promoted the NRF2 pathway in RNF113A KO cells compared to that in WT cells. Moreover, we found that RNF113A KO reduced the ROS-protective activities.

In response to oxidative stress, virus infection, endoplasmic reticulum (ER) stress, and nutrient depletion, PKR, HRI, PERK, and GCN2 are activated, leading to phosphorylation of eIF2α for repressing protein translation and protecting mRNA by SG formation (Lavalee et al. 2021; Jung et al. 2023). After confirming an increase in ROS levels in RNF113A KO cells, we observed a significant increase in SG formation. We believe that the reduction in ROS-scavenging activity by RNF113A KO may increase arsenite-induced SG formation. Further research to identify the correlation between SG formation and RNF113A deficiency-induced oxidative stress will provide new insights into the pathogenesis of RNF113A-associated diseases in humans.

One of the limitations of our study is that we analyzed the roles of RNF113A only in the HeLa cell line. Patients with TTD exhibit defects in various tissues, including the brain and skin (Brooks et al. 2011). Therefore, the specific model cell lines for study the TTD still unclear. In this study, we KO RNF113A in the HeLa cell line, which is widely used as a model for exploring DNA repair mechanisms. However, additional studies identifying the role of RNF113A in various tissue-oriented cell lines, as well as in animal models, will be required to understand the detailed mechanism contributing to RNF113A-associated human diseases. Another limitation is that we did not prove the direct function of the RNF113A protein in regulating oxidative stress and SG formation. We speculate that RNF113A may affects widely on the transcriptome and proteome through its roles as spliceosome component, DNA damage repair factor, and E3 ubiquitin ligase, potentially leading to oxidative stress in cells (Brickner et al. 2017; Tsao et al. 2021; Yang et al. 2023). However, additional studies are required to elucidate the detailed mechanisms, from the direct role of the RNF113A protein to the global alteration of NRF2 pathway target genes.

Here, we found the activation of the NRF2 pathway in RNF113A KO HeLa cells and also confirmed the strong nuclear localization of the RNF113A protein through immunofluorescence analysis. Based on these finding, it could be speculated that the RNF113A protein may indirectly affect the nuclear translocation of the NRF2 protein, rather than directly interacting with the NRF2-KEAP1 protein complex. Intracellular ROS level is an inducer of the NRF2 pathway, and RNF113A deficiency-promoted increase of intracellular ROS level may induce the NRF2 pathway. Furthermore, although the NRF2 pathway serves as a defense mechanism protecting from cellular stresses, including oxidative stress, RNF113A KO HeLa cells exhibited high intracellular ROS levels and were sensitized to H_2_O_2_-induced cell death. These results suggest that the deficiency of RNF113A causes oxidative stress, leading to the activation of the NRF2 pathway to maintain redox homeostasis. RNF113A-deficient cells may nevertheless be susceptible to oxidative stress-induced cell death. Further studies demonstrating the exact roles of RNF113A in regulating oxidative stresses could be helpful in understanding cellular oxidative stress defense systems.

Our results could provide useful information for identifying the mechanisms underlying human diseases associated with RNF113A deficiency. A nonsense mutation in the *RNF113A* gene, from cytosine to thymine at position 901, was identified through pedigree analysis of a family with X-linked TTD manifestations in some members (Corbett et al. 2015). However, the detailed mechanisms of RNF113A deficiency in developing X-linked TTD remain unclear. Recent studies have identified RNF113A as a component of the spliceosome complex based on structural analysis using cryo-EM (Haselbach et al. 2018 Fica et al. 2019;). Moreover, alternative splicing analysis revealed that RNF113A deficiency affects global RNA splicing (Shostak et al. 2020). Other studies have focused on the function of RNF113A in the repair of DNA alkylation damage (Brickner et al. 2017; Tsao et al. 2021; Lukinovic et al. 2022). However, the effect of RNF113A deficiency on intracellular ROS levels has not been well-studied. Our results provide new information on the cellular effects in RNF113A KO, particularly focusing on oxidative stress responses and the NRF2 pathway. Patients with TTD are characterized by a premature aging phenotype and a defect in DNA repair systems, both strongly associated with oxidative stresses (Faghri et al. 2008). Intracellular ROS increase the instability of genomic DNA, leading to aging phenotypes (Oh et al. 2014; Park et al. 2020). Further experiments identifying the synergistic effects of DNA instability and oxidative stress on RNF113A deficiency cells may offer new clues to identifying TTD pathogenesis. Moreover, impaired DNA damage repair pathways and dysregulation of oxidative stress are recognized as crucial drivers of cancer development (Cheung and Vousden 2022). A recent study identified the upregulation of RNF113A in esophageal squamous cell carcinoma compared with adjacent normal tissues and its roles in oncogenic properties (Wang et al. 2018). Further research should determine the impacts of DNA repair activities and cellular protective roles of RNF113A in cancer development and progression. We believe that our RNA sequencing analysis of RNF113A KO cells and the identification of the roles of RNF113A in ROS protective activities provide valuable insights into the potential role of RNF113A in human diseases, including X-linked TTD and cancer.

## Supplementary Material

Supplemental Material

## Data Availability

The data that support the findings of this study are available from the corresponding author upon reasonable request. Raw RNA sequencing data are deposited in the NCBI SRA database with the accession numbers SRR28554989-SRR28554992 under NCBI BioPrjoect PRJNA1096255.
