# Supplementary material for: Effect of RNF113A deficiency on oxidative stress-induced NRF2 pathway
Source: Anim Cells Syst (Seoul). 2024 May 11;28(1):261–71. doi: 10.1080/19768354.2024.2349758 (PMC11089925; doi:10.1080/19768354.2024.2349758)
Supplement: Supplemental Material [file TACS_A_2349758_SM2291.docx]

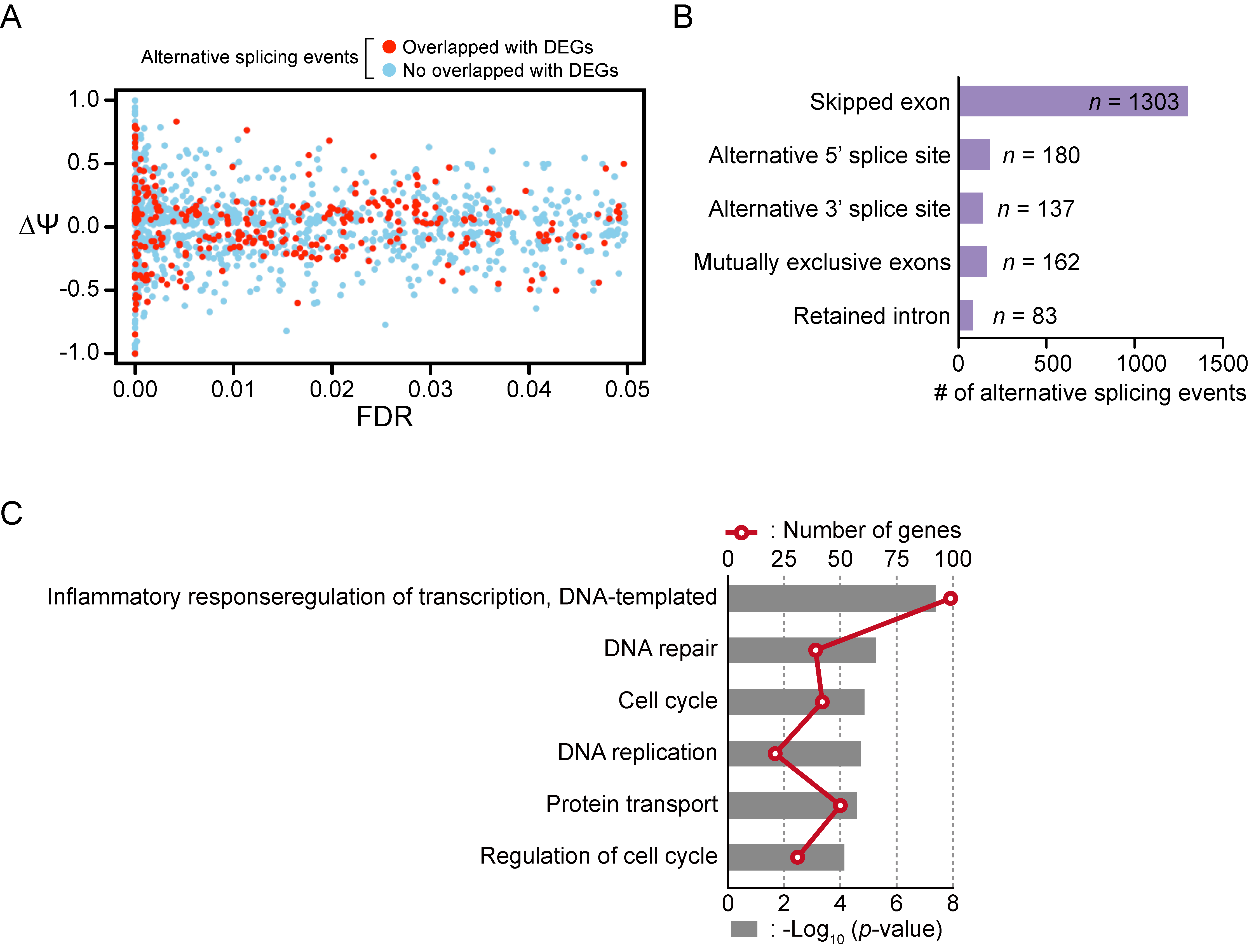


**Supplemental Figure 1.** Comprehensive analysis of alternative splicing in RNF113A KO HeLa cells. (A) Scatter plot showing the differential alternative splicing events between WT and RNF113A KO cells. (B) Bar graph showing the number events of each type of alternative splicing. (C) Gene Ontology enrichment analysis of biological process for alternatively spliced genes in RNF113A KO HeLa cells versus WT HeLa cells.
